# Stacked probability plots of the extended illness-death model using constant transition hazards – an easy to use shiny app

**DOI:** 10.1186/s12874-024-02240-3

**Published:** 2024-05-18

**Authors:** Marlon Grodd, Susanne Weber, Martin Wolkewitz

**Affiliations:** 1 Institute of Medical Biometry and Statistics, Medical Center - University of Freiburg, Faculty of Medicine, Freiburg, Germany; 2 Freiburg Center for Data Analysis and Modeling, University of Freiburg, Freiburg, Germany

**Keywords:** Markov process, Transition probability, Hazard rates, R, Extended illness-death model, Stacked probability plot, Shiny app

## Abstract

**Background:**

Extended illness-death models (a specific class of multistate models) are a useful tool to analyse situations like hospital-acquired infections, ventilation-associated pneumonia, and transfers between hospitals. The main components of these models are hazard rates and transition probabilities. Calculation of different measures and their interpretation can be challenging due to their complexity.

**Methods:**

By assuming time-constant hazards, the complexity of these models becomes manageable and closed mathematical forms for transition probabilities can be derived. Using these forms, we created a tool in R to visualize transition probabilities via stacked probability plots.

**Results:**

In this article, we present this tool and give some insights into its theoretical background. Using published examples, we give guidelines on how this tool can be used. Our goal is to provide an instrument that helps obtain a deeper understanding of a complex multistate setting.

**Conclusion:**

While multistate models (in particular extended illness-death models), can be highly complex, this tool can be used in studies to both understand assumptions, which have been made during planning and as a first step in analysing complex data structures. An online version of this tool can be found at https://eidm.imbi.uni-freiburg.de/.

**Supplementary Information:**

The online version contains supplementary material available at 10.1186/s12874-024-02240-3.

## Background

Choosing and understanding statistical analysis models in epidemiology can be challenging. Many models have distinct shortcomings. For example, standard logistic regression ignores the timing of events and therefore, only provides a restricted view. Kaplan-Meier models take into account the timing of events but fail to consider competing events. Analysing hospital-acquired infections (HAI), ventilation-associated pneumonia (VAP), worsening of COVID19-cases in hospital or transfers of COVID19-ICU-cases are just a few examples of situations where competing events like death and discharge have to be taken into account.

The European Medicines Agency (EMA) has included the occurrence of intercurrent events in its list of things to consider when describing treatment effects [1]. Multistate models are one way to meet this requirement. U. Beyer et al. [2] and A. Erdmann et al. [3] showed the use of multistate models to address the occurrence of intermediate events in cancer patients with a slightly different models.

Multistate (especially competing risk) models are becoming a more and more established tool to analyse such complex settings. Many authors have already pointed out the importance of being careful in the presence of competing events [4, 5] and have given suggestions on which methods to use in specific situations [6–9]. C. H. Jackson et al. [10] shows the advantage of multistate models by comparing different modelling frameworks in a model which is quite similar to the one used in this article.

**Fig. 1:**
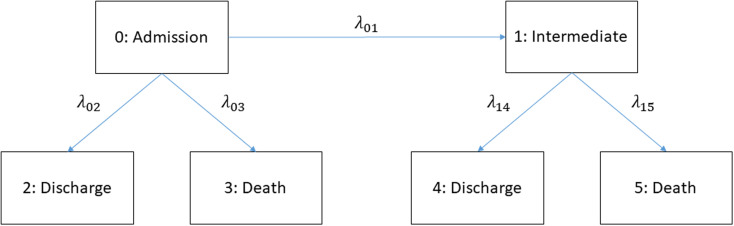
Extended illness-death model with constant hazard rates

In this work we will focus on the extended illness-death model (eidm) which can be seen in Fig. 1 and is described in detail in [6] while considering constant hazards. The implied limitations will be discussed in the conclusion section. This model distinguishes between two absorbing events before or after the intermediate event. The term “intermediate event” depends on the setting of the study. Some examples could be disease progression, as in the articles by U. Beyer et al. [2] and A. Erdmann et al. [3] or nosocomial pneumonia like seen in the work of B. François et al. [11] and J. Chastre et al. [12]. This accounts for the time-dependencies of intermediate events like HAI, VAP, and worsening patient condition or transfer of COVID-19-cases.

Using constant transition hazards offers the opportunity to calculate closed forms of transition probabilities at a given point in time. The second advantage of using the constant hazard framework is that there are few to no data requirements. These transition probabilities can be visualized in stacked probability plots which are discussed in detail by Hazard et al. and von Cube et al. [15, 13].

Our goal is to provide a tool, which utilizes the benefits of a multistate model especially in the framework of constant hazards and the visual advantages of stacked probability plots to provide a tool for non-statisticians like clinicians to improve the planning and analysis of epidemiologic studies. Specifically, for studies with non-mortal Endpoints (e.g. discharge or infection) corresponding to an extended illness-death model. Hence, we implemented an app using R [14]. This app takes hazards or hazard ratios as inputs and renders corresponding stacked probability plots, plots for the population attributable fraction (PAF), and plots for attributable mortality (AM) (not discussed in this article).

In the following, we present statistical considerations for our calculations followed by a guideline on how to use this tool. In addition, we give some hypothetical and real examples.

## Implementation

We consider a finite state continuous time markov process $X\left(t\right)$ which can occupy states in $\{0,\dots ,5\}$ at a given time $t$, see Fig. 1. Since this process is markov, the transition probabilities (i.e. the probability to be in state $j$ at time $t$ while previously being in state $i$ at time $s$) can be written as

$${P}_{ij}\left(s,t\right)=P\left(X\left(t\right)=j|X\left(s\right)=i\right).$$

By considering every possible transition from state $i$ to state $j$, we get a transition probability matrix $P\left(t\right)$ given by:

$$P\left(t\right)={\left({P}_{ij}\left(0,t\right)\right)}_{i,j}=P\left(0,t\right)$$

$$= \left(\begin{array}{ccc}{P}_{11}(0, t)& \cdots & {P}_{15}(0, t)\\ \vdots & \ddots & \vdots \\ {P}_{51}(0, t)& \dots & {P}_{55}(0, t)\end{array}\right)$$

**Fig. 2:**
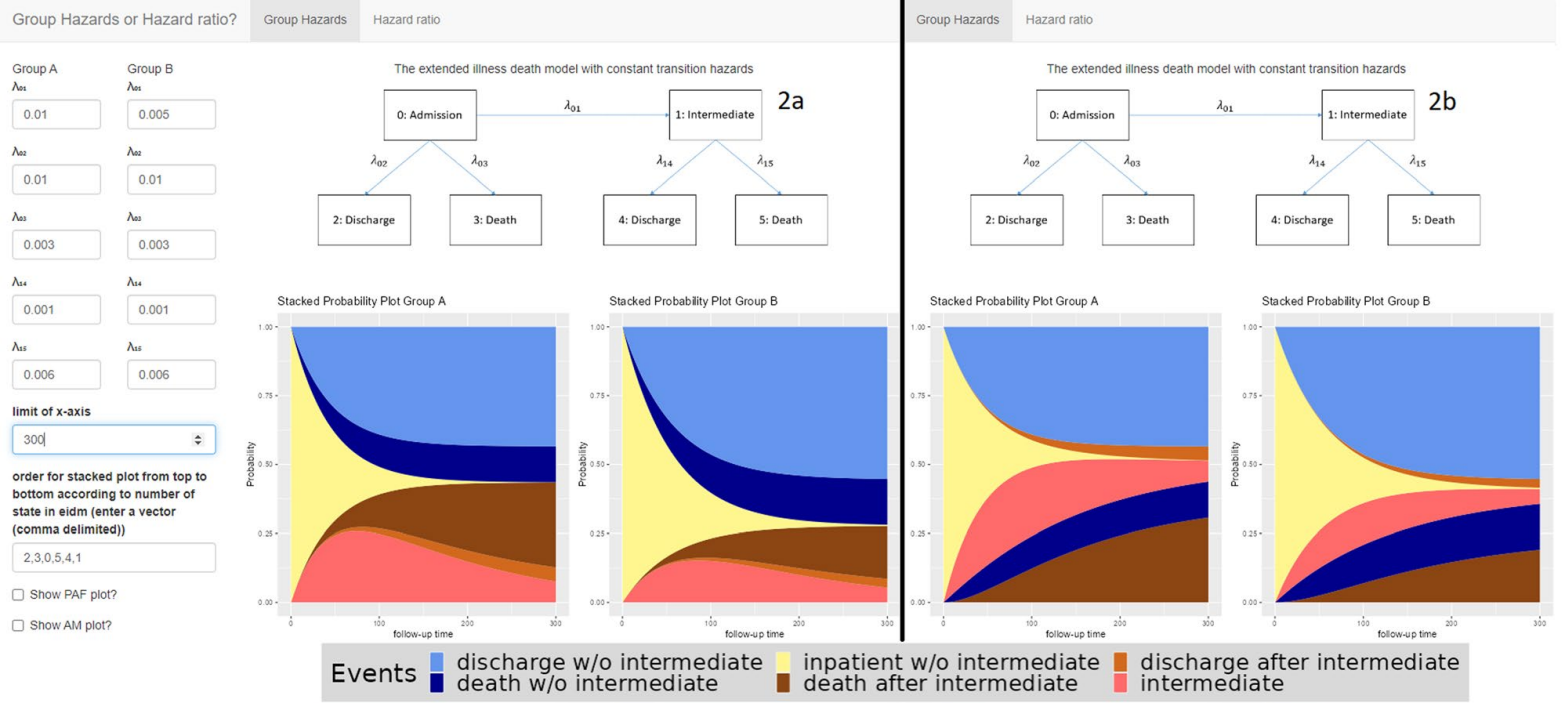
Main window and output of the tool for example 1

**Fig. 3:**
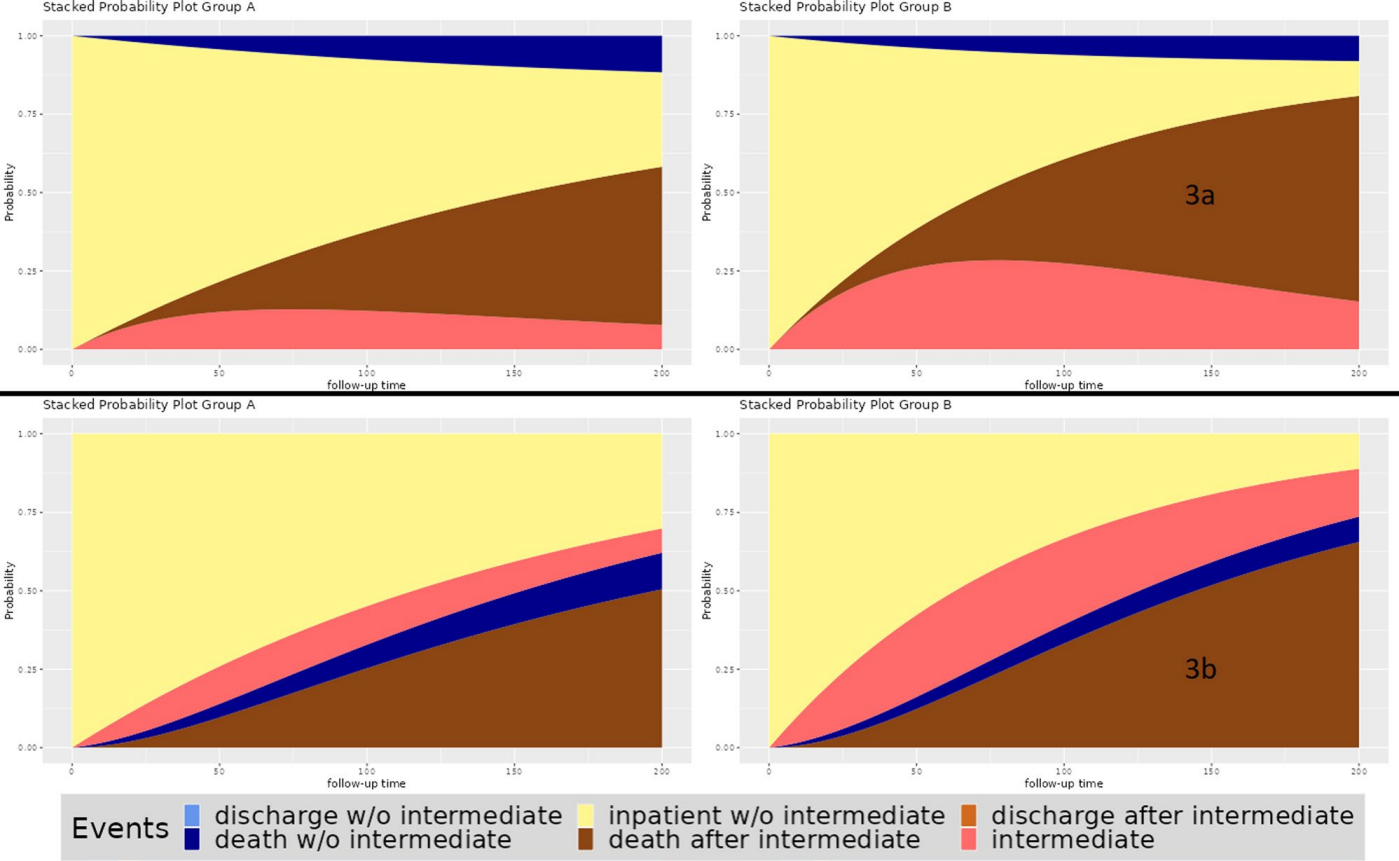
Output of the tool from the second example

The explicit formulas for those probabilities can be found in the supplement material. Those formulas can be used to plot the transition probabilities ${P}_{ij}(0,t)$ dependent on the hazards ${\lambda }_{ij}$. Finally, we stack these probability plots upon each other to create a stacked probability plot. See Figs. 2 and 3 for some example visualizations.

Therefore, by plotting those probabilities over time stacked on each other, we get a graphic that ranges from $0$ to $1$ on the y-Axis, hence each Probability is represented by a specific area in this plot. Those areas can be interpreted as the time spent in certain state [15]. Arranging those areas can furthermore help to interpret the sum of specific areas as cumulative incidence i.e. ${P}_{01}\left(0,t\right)+ {P}_{04}\left(0,t\right)+ {P}_{05}\left(0,t\right)$ is the cumulative probability to get an intermediate event until time $t$.

## Application

In order to use this tool, one first needs to estimate time constant hazard rates. These are calculated by dividing the number of transitions by the time at risk in the state from which the transition occurs:

$${\lambda }_{01}= \frac{{N}_{01}}{{T}_{0}}\quad { \lambda }_{02}= \frac{{N}_{02}}{{T}_{0}}\,\quad{ \lambda }_{03}= \frac{{N}_{03}}{{T}_{0}} \quad{\lambda }_{14}= \frac{{N}_{14}}{{T}_{1}}\ \quad{ \lambda }_{15}= \frac{{N}_{15}}{{T}_{1}}$$

Where ${N}_{ij}$ corresponds to the number of transitions from state *i* to state *j* and ${T}_{i}$ is the total time at risk in state $i$.

These hazard rates can be plugged into the tool. In the following we discuss some examples to illustrate the application.

The absolute easiest and intended way to actually use this tool is to go to https://eidm.imbi.uni-freiburg.de/ and start playing around.

If you are interested in using this tool locally, it gets more technical. The necessary code and instructions can be found in the supplementary material or at https://github.com/marlongrodd/eidm.

Figure 2 shows the interface of the application. First there are some instructions on how to use the tool. Then you can choose to enter either the hazards for each group separately, or just the hazards for group A and the corresponding hazard ratios for group B. The next input fields are for the hazards and hazard ratios, depending on the choice made earlier. The “Limit of x-axis” option can be used to limit the plots to certain time periods. The ‘Order of stacked plot’ field is used to determine the order in which the coloured areas are stacked. Finally, you can choose to display the PAF and AM plots as well.

## Examples

Multistate models, and in particular extended illness-death models, can have beneficial insights into the structural background of many situations. For instance, some examples in the hospital setting are listed in Table 1.

Note that in this work we focus on the hospital setting. However, there are many other settings that can be analyzed using multistate models. These models are relevant as soon as there are several outcomes of interest or intermediate events. For instance disease progression as an intermediate event for outpatients. In the following, some of the examples in a hospital setting loosely referring to real world data will be presented. We take a closer look at examples for ventilation-acquired infections and disease progression. Aside from describing the usage and interpretation of the stacked probability plots, one further focus will be on how one can order the plot areas.

**Table 1:** Examples of initial states, intermediate events and absorbing states for possible applications

Initial state	Intermediate state	Absorbing states
Hospitalized	Hospital-acquired infection (HAI) ICU admission Transfers between hospitals Disease progression Decubitus Delirium	Death Discharge
ICU	Starting ventilation Disease progression Decubitus	Death Discharge ICU Discharge
Ventilated	Ventilation-associated infection Extubation after ventilation Disease progression Decubitus	Death Discharge Extubation after ventilation ICU Discharge

Three examples are considered. The corresponding hazards are given in Table 2.

**Table 2:** Hazards for the examples

	Example 1		Example 2		Example 3
	VAP		Disease progression		Real data example *N* = 756
Group	A	B	A	B	
${\lambda }_{01}$	0.01	0.005	0.005	0.01	0.01924868
${\lambda }_{02}$	0.01	0.01	0	0	0.07373486
${\lambda }_{03}$	0.003	0.003	0.001	0.001	0.02437131
${\lambda }_{14}$	0.001	0.001	0	0	0.03453569
${\lambda }_{15}$	0.006	0.006	0.02	0.015	0.01304682

VAP = Ventilation-associated pneumonia, Real data example: random sample of the SIR-3 Dataset found in the “etm” R package [16]

### Example 1

considers VAP as an intermediate event. The only effect of the group variable is on the hazard of the transition from the initial state to VAP (decreasing hazard). The question of interest is how does this single difference effect the occurrence of all possible transitions?

### Example 2

considers a completely different setting with disease progression as intermediate event and only one absorbing state (death). There is an increased hazard for the intermediate event and a decreased hazard for death after the intermediate event in group B compared to group A. How do these two effects in different directions affect the overall occurrence of death?

### Example 3

illustrates the differences between the full follow-up analysis and the simplified constant hazard approach. Real data of hospitalized patients is considered. Hospital acquired infection is the intermediate event and discharge or death are the absorbing events.

Note that if a transition hazard is zero this means that this transition is not possible. Thus, example 2 reduces to a simple illness-death model with one intermediate and only one absorbing state (death).

For a detailed description of the examples, see the following section.

### Example 1: Ventilation-associated pneumonia

In this example, we consider ventilated patients and VAP as intermediate event. Motivated by the project EVADE (Effort to Prevent Nosocomial Pneumonia caused by Pseudomonas aeruginosa in Mechanically ventilated Subjects) which is part of the COMBACTE (Combatting Bacterial Resistance in Europe, https://www.combacte.com/) consortium, we discuss the effects of VAP on death and discharge [12]. The aim of this project was to analyze the impact of treatment with specific antibodies in ventilated patients against VAP. Therefore, information about observation times of VAP, death and discharge have been collected. Note that our focus lies in presenting the results. Our aim is not to discuss medical implications. The hazards are not the real hazards from the trial, but are inspired by the real hazards calculated from the dataset by dividing the number of events by the number of patient days in hospital. Those hazards can be found in Table 2. Using those values as input, the tool provides the plots as presented in Fig. 2a.

Group A corresponds to the control group and group B to the intervention group. Note that the death and discharge hazards are the same in both treatment groups. Only the VAP hazard (${\lambda }_{01}$) differs between the treatment groups and is lower in group B compared to group A, implying an advantage in group B with respect to VAP. However, the death and discharge hazards before the intermediate event differ from the death and discharge hazard after the intermediate event (equal hazards in both groups). The hazard for death is lower before VAP compared to the time afterwards (${\lambda }_{03}$compared to ${\lambda }_{15}$). In contrast the discharge hazard before VAP is greater than afterwards (${\lambda }_{02}$ compared to ${\lambda }_{14}$).

Hence, as the probability of the intermediate event differs between treatment groups, the overall probabilities of dying and being discharged with and without the treatment differ too, even if there is no difference in the death and discharge hazards between the groups. The overall probability of dying declines after receiving the treatment. For discharge, this effect can be seen in the opposite direction. This effect can also be highlighted using the tool, see Fig. 2b. In this figure, the order of the areas is modified. On the top of Fig. 2b, the two desirable discharge states are combined. At the bottom, the three non-desirable states are plotted. The dark blue and dark brown area represent the death states (without and with VAP) and thus overall mortality. These combined areas on the left hand side is lower than those on the right hand side. Meanwhile the combined areas for discharge (without or with VAP) at the top of the plot is greater on the right than on the left graphics.

### Example 2: Disease progression

Our second example considers disease progression as an intermediate event and orientates on [17]. The focus of this study was to analyse the effect of the drug Selexipag on the occurrence of complications related to pulmonary hypertension. This article provides information about the total number of patients, the number of disease progressions, deaths and median follow up. With these numbers, we can calculate the corresponding transition hazards by dividing the number of events (progression or death) by the patient days. In fact the numbers used in our example are just inspired by those given in the paper. We ignore discharge in this example and consequently use a simple illness-death model. The resulting plots are given in Fig. 3a and b, considering different orders.

The hazard rates for discharge are set to zero, making this transition not possible and reducing the model to a simple illness-death model. Consequently, there are no areas present for discharge in this plot. The hazard for complications related to pulmonary hypertension (intermediate event) is higher in group B compared to group A. Thus, the area for the intermediate event is bigger on the right side. The death-hazard before complications related to pulmonary hypertension is the same in both groups. However, the intermediate event leads to a higher death hazard in both groups. This increase is higher in group A. Thus, on the one hand, Group B has a disadvantage concerning the intermediate event, but on the other hand has an advantage concerning death after intermediate event. This leads to the situation that the two differences in hazards basically cancel each other out. If the death-hazard after the intermediate event would be the same in both groups the higher hazard for the intermediate event would lead to a much higher probability of dying in general in group B (blue and brown area combined).

### Example 3: Real data

This example uses the los.data dataset from the R package “etm” to compare the constant hazard approach with non-parametric estimation using the Aalen-Johansen estimator [18].

The los.data consists of a sample of the dataset from the SIR-3 study, an observational cohort study to analyse the burden of hospital-acquired infections [16].

**Fig. 4:**
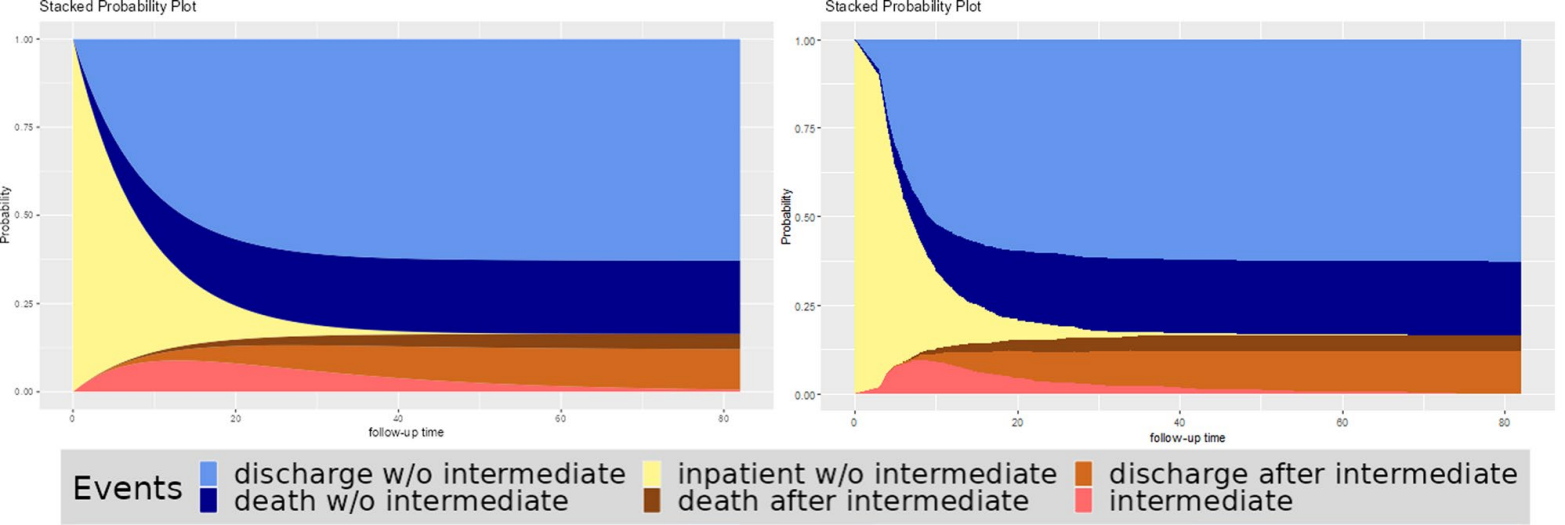
Comparison constant hazard (left) with non-parametric estimation using the etm package in R (right)

As this dataset does not distinguish between different groups, we will focus on the differences between the models. We calculated constant transition hazards for the five possible transitions (baseline -> discharge, death or intermediate event; intermediate event -> death or discharge) and used these in the application by dividing the number of events by the total amount of time patients spend in each state (see section “Application” for more details). In addition, we used the etm function to calculate empirical state occupancy probabilities and plotted these using the R package ggplot2. We arranged these two plots together for better comparison (see Fig. 4).

It can be seen that the use of constant hazard can mimic the empirical state occupancy probabilities. Thus, if there are no real data but information about patient days and number of events for each transition, one can calculate the corresponding hazard rates and use them to get a good idea of what the probabilities from the real data set might look like.

On the other hand, there are clearly some differences. In this example, the first days differ between these two plots; while the intermediate event has a high slope in the constant hazard approach, this behaviour is not seen in the empirical estimate, where the high slope does not start until day four. This is an obvious violation of the constant hazard assumption and shows that this tool should not be used for the final analysis of the data, but rather to get an impression of the possible results of planned studies.

## Conclusion

In conclusion, this tool can be used to translate the transition hazards into probabilities and furthermore to visualize the impact of a single varying hazard on all transition probabilities.

Furthermore, one can investigate the impact of different hazards with both, desirable and non-desirable effects. Additionally, one can experience the impact of varying hazard rates on the probabilities (i.e. what would happen if in example 1 the hazards for death before and after intermediate were also impacted by the intervention? What are the Probabilities if the intervention does affect the hazard for discharge rather than the hazard for the intermediate event? ).

Investigation of effects when dealing with intermediate events faces different challenges. These settings can be analysed using multistate models. However, events and effects depend on time and all possible hazard rates. This creates a level of complexity that adds difficulty to achieving proper interpretation, planning, and analysis of epidemiologic studies. In this article, a tool to visualize multistate models in an extended-illness-death-setting was discussed. This is a common setting in epidemiologic studies addressing an intermediate event, e.g. studies on nosocomial infections [12].

This tool provides the benefits of multistate models and facilitates the interpretation of complex correlation. By visualizing the impact of expected hazard ratios in specific scenarios, one can acquire a better understanding of the effects of their intervention.

Furthermore, the area between the curves can be considered as the expected length of stay in the respected state. For example, Fig. 3 group B stays much longer in the initial state (yellow area) compared to group A. In contrast, the time “spent” in the state “death after intermediate” (brown area) is greater for group A since the corresponding area is greater. The expected length of stay in the state “intermediate” without further progression is comparable in both groups.

Additionally, three examples were used to exhibit possible applications of this tool. In the first two examples, we orientated on the COMBACTE-study EVADE and discussed the direct and indirect impacts of an intervention on intermediate events, death and discharge. The second example considered pulmonary hypertension [17] to cover settings where one terminal event, e.g. hospital discharge, is not present.

It is important to note that the assumption of constant hazards is a huge simplification and limitation of this tool. Constant hazards imply that the underlying mechanisms are independent of time. This is usually not the case in practice, so this tool should be used during the design phase of a study, or at most as a first step in the process of data analysis. For more complex analyses, R packages such as etm [18] or mstate [19] are more appropriate. How to use multistate models to analyse data sets from epidemiological studies is described in more detail in R. J. Cook & J. F. Lawless [20] in A. Bühler et al. [21] in J. Beyersmann et al. [22] or in P. Hougaard [23]. C. H. Jackson [10] showed the use of a similar model applied to outcomes after admission with COVID-19 in two frameworks (transition-specific hazard functions and mixture multi-state models) of parametric models using gamma distributions.

Our aim was to provide a tool to simplify the complexity of multi-state models and to promote a better understanding of these processes. An additional concern was to give this tool a wide range of possible applications; Table 1 gives some examples of possible settings in a hospital environment, without claiming to be exhaustive. Extended illness-death models can be applied whenever there is an intermediate event and a final event of interest other than death.

In further steps, our tool could be extended to more different models, as we see for example in M. Lafuente et al.‘s [24] established stand-alone tool for visualisation and prediction of multistate processes on ICU occupancy by patients with COVID-19.

The R-code for this tool is provided in the supplement file “Additional file 1.docx” as well as on https://github.com/marlongrodd/eidm.

Availability and requirements.

- Project name: Extended illness-death model with constant transition hazards.
- Project home page: https://eidm.imbi.uni-freiburg.de/.
- Operating system(s): Platform independent.
- Programming language: R.
- Other requirements: R version 4.2.2 or higher.
- License: GNU GPL.

### Electronic supplementary material

Below is the link to the electronic supplementary material.

Supplementary Material 1

## Data Availability

Data sharing is not applicable to this article as no datasets were generated or analysed during the current study.

## Abbreviations

HAI: Hospital-acquired infections
VAP: Ventilation-associated pneumonia
Eidm: Extended illness-death model
