# Supplementary material for: Stacked probability plots of the extended illness-death model using constant transition hazards – an easy to use shiny app
Source: BMC Med Res Methodol. 2024 May 18;24:116. doi: 10.1186/s12874-024-02240-3 (PMC11102298; doi:10.1186/s12874-024-02240-3)
Supplement: Supplementary file 1 — Supplementary Material 1 [file 12874_2024_2240_MOESM1_ESM.docx]

# Additional file 1.docx:

# Explicit probability formulas

$P_{ij}(0,t)$ can be expressed in explicit formulas as demonstrated by Cube et al (6). With

$$\lambda_{0}=\lambda_{01}+\lambda_{02}+\lambda_{03}$$

$$\lambda_{1}= \lambda_{14}+\lambda_{15}$$

the transition probabilities can be expressed as follows:

$$P_{00}\left( 0, t \right)=e^{-\lambda_{0}\cdot t}$$

$$P_{11}\left( 0, t \right)=e^{-\lambda_{1}\cdot t}$$

$$P_{01}\left( 0, t \right)=\left\{ \begin{aligned} \frac{\lambda_{01}}{\lambda_{1}- \lambda_{0}}\left( e^{-\lambda_{0}\cdot t}-e^{-\lambda_{1}\cdot t} \right) if \lambda_{1}\neq\lambda_{0} \\ \lambda_{01}\cdot e^{-\lambda_{1}\cdot t}\cdot t if \lambda_{1}=\lambda_{0} \end{aligned} \right.$$

$$P_{02}\left( 0, t \right)=\frac{\lambda_{02}}{\lambda_{0}}\left( 1-e^{-\lambda_{0}\cdot t} \right)$$

$$P_{03}\left( 0, t \right)=\frac{\lambda_{03}}{\lambda_{0}}\left( 1-e^{-\lambda_{0}\cdot t} \right)$$

$$P_{14}\left( 0, t \right)=\frac{\lambda_{14}}{\lambda_{1}}\left( 1-P_{11}(0, t) \right)= \frac{\lambda_{14}}{\lambda_{1}}\left( 1-e^{-\lambda_{1}\cdot t} \right)$$

$$P_{15}\left( 0, t \right)=\frac{\lambda_{15}}{\lambda_{1}}\left( 1-P_{11}(0, t) \right)= \frac{\lambda_{15}}{\lambda_{1}}\left( 1-e^{-\lambda_{1}\cdot t} \right)$$

$$P_{04}\left( 0,t \right)=\frac{\lambda_{14}}{\lambda_{1}}\left( 1-P_{00}\left( 0,t \right)-P_{01}\left( 0,t \right)-P_{02}\left( 0,t \right)-P_{03}\left( 0.t \right) \right)=\left\{ \begin{aligned} \frac{\lambda_{01}\cdot\lambda_{14}}{\lambda_{0}\cdot\lambda_{1}}-\frac{\lambda_{01}\cdot\lambda_{14}}{\lambda_{0}\cdot\left( \lambda_{1}- \lambda_{0} \right)}\left( e^{-\lambda_{1}\cdot t}-e^{-\lambda_{0}\cdot t} \right) if \lambda_{1}\neq\lambda_{0} \\ \frac{\lambda_{01}\cdot\lambda_{14}}{\lambda_{0}\cdot\lambda_{1}}-\frac{\lambda_{01}\cdot\lambda_{14}}{\lambda_{0}\cdot\lambda_{1}}e^{-\lambda_{0}\cdot t}-\frac{\lambda_{01}\cdot\lambda_{14}}{\lambda_{1}}\cdot e^{-\lambda_{1}\cdot t}\cdot t else \end{aligned} \right.$$

$$P_{05}\left( 0,t \right)=\frac{\lambda_{15}}{\lambda_{1}}\left( 1-P_{00}\left( 0,t \right)-P_{01}\left( 0,t \right)-P_{02}\left( 0,t \right)-P_{03}\left( 0.t \right) \right)=\left\{ \begin{aligned} \frac{\lambda_{01}\cdot\lambda_{15}}{\lambda_{0}\cdot\lambda_{1}}-\frac{\lambda_{01}\cdot\lambda_{15}}{\lambda_{0}\cdot\left( \lambda_{1}- \lambda_{0} \right)}\left( e^{-\lambda_{1}\cdot t}-e^{-\lambda_{0}\cdot t} \right) if \lambda_{1}\neq\lambda_{0} \\ \frac{\lambda_{01}\cdot\lambda_{15}}{\lambda_{0}\cdot\lambda_{1}}-\frac{\lambda_{01}\cdot\lambda_{15}}{\lambda_{0}\cdot\lambda_{1}}e^{-\lambda_{0}\cdot t}-\frac{\lambda_{01}\cdot\lambda_{15}}{\lambda_{1}}\cdot e^{-\lambda_{1}\cdot t}\cdot t else \end{aligned} \right.$$

Note that $P_{00}\left( 0, t \right)+ P_{01}\left( 0, t \right)+ P_{02}\left( 0, t \right)+ P_{03}\left( 0, t \right)+ P_{04}\left( 0, t \right)+ P_{05}\left( 0, t \right)=1$ for every $t$.

# Program Code

Here you can find the code for the application. This code can also be found at https://github.com/marlongrodd/eidm. R version 4.3.1 or higher is required (lower versions not tested). The scripts must be placed in the same folder and each must have it's own file named like the specific headline.

Open the file "run.R". Lines 20 to 25 of this script list the packages that need to be installed to run the tool. You can now choose the working directory by simply pointing to it in line 34, or use the "choose.dir" function provided by R (does not work on all systems). Finally, to run the application, simply execute the whole "run.R" script. If you choose to use "choose.dir", the script should ask for a directory location. This must be the directory where all the scripts are located.

## run.R

#-------------------------------------------------------------------

#

# Script for running the App via shiny-server

#

# Copyright (C) 2023 Marlon Grodd, Susanne Weber, Martin Wolkewitz

#

# This program is free software: you can redistribute it and/or modify

# it under the terms of the GNU General Public License as published by

# the Free Software Foundation, either version 3 of the License, or

# (at your option) any later version.

#

# This program is distributed in the hope that it will be useful,

# but WITHOUT ANY WARRANTY; without even the implied warranty of

# MERCHANTABILITY or FITNESS FOR A PARTICULAR PURPOSE. See the

# GNU General Public License for more details.

#

# You should have received a copy of the GNU General Public License

# along with this program. If not, see <http://www.gnu.org/licenses/>.

#

# Neseccary Packages

# install.packages("dplyr")

# install.packages("ggplot2")

# install.packages("gridExtra")

# install.packages("expm")

# install.packages("shiny")

#

#-------------------------------------------------------------------

library(shiny)

#--------------------------------- if you run local ----------------

setwd(choose.dir()) # choose the folder of this script

# setwd("put/path/here") # choose the folder of this script

runApp()

## app.R

#-------------------------------------------------------------------

#

# script for starting the app

#

# Copyright (C) 2023 Marlon Grodd, Susanne Weber, Martin Wolkewitz

#

# This program is free software: you can redistribute it and/or modify

# it under the terms of the GNU General Public License as published by

# the Free Software Foundation, either version 3 of the License, or

# (at your option) any later version.

#

# This program is distributed in the hope that it will be useful,

# but WITHOUT ANY WARRANTY; without even the implied warranty of

# MERCHANTABILITY or FITNESS FOR A PARTICULAR PURPOSE. See the

# GNU General Public License for more details.

#

# You should have received a copy of the GNU General Public License

# along with this program. If not, see <http://www.gnu.org/licenses/>.

#

#-------------------------------------------------------------------

require(dplyr)

require(ggplot2)

require(gridExtra)

require(expm)

source("functions.R")

source("interface.R")

shinyApp(ui, server)

## functions.R

#-----------------------------------------------------------------------------

#

# Analysis of extended illness-death model

#

# Copyright (C) 2023 Marlon Grodd, Susanne Weber, Martin Wolkewitz

#

# This program is free software: you can redistribute it and/or modify

# it under the terms of the GNU General Public License as published by

# the Free Software Foundation, either version 3 of the License, or

# (at your option) any later version.

#

# This program is distributed in the hope that it will be useful,

# but WITHOUT ANY WARRANTY; without even the implied warranty of

# MERCHANTABILITY or FITNESS FOR A PARTICULAR PURPOSE. See the

# GNU General Public License for more details.

#

# You should have received a copy of the GNU General Public License

# along with this program. If not, see <http://www.gnu.org/licenses/>.

#

# 0: admission

# 1: intermediate event

# 2: discharge w/o intermediate event

# 3: death w/o intermediate event

# 4: discharge after intermediate event

# 5: death after intermediate event

#

# Function input:

# aggregated transition hazards

#

# Function output:

# transition probabilities

# AM, PAF

# cLOS

#

# returned Plots:

# AM, PAF

# Stacked plots:

# eidm

#

# structure of code:

#

# define two functions:

# 1) transition_probs: calculation of Pij, PAF, AM, clos

# 2) stacked_plot_eidm: create stacked plots (use results of 1)

#

# -> defining function combining previous functions

#

#-----------------------------------------------------------------------------

#-----------------------------------------------------------------------------

# (1)

# Cube, M., Schumacher, M., Wolkewitz, M. (2017). Basic parametric analysis for a multi-state

# model in hospital epidemiology. Pages: 3-5. In: BMC Meidcal Research Methodology.

# (2)

# Cube, M., Schumacher, M., Wolkewitz, M. (2017). Multistate Modeling to Analyze Nosocomial

# Infection Data: An Introduction and Demonstration. Pages: 955-956.

# In: infection control & hospital epidemiology.

#

#-----------------------------------------------------------------------------

#-----------------------------------------------------------------------------

# transition probability function for eidm

#-----------------------------------------------------------------------------

#---------------

# Function 1.

#---------------

transition_prob <- function(t,

Lambda_01,

Lambda_02,

Lambda_03,

Lambda_14,

Lambda_15) {

# Hazard Matrix

Q <- matrix(0,

nrow = 6,

ncol = 6)

Q[1, 2] <- Lambda_01

Q[1, 3] <- Lambda_02

Q[1, 4] <- Lambda_03

Q[2, 5] <- Lambda_14

Q[2, 6] <- Lambda_15

Q[1, 1] <- -sum(Q[1, ])

Q[2, 2] <- -sum(Q[2, ])

# Probability Matrix

P_0J <- expm(Q * t)[1, ]

P00 <- P_0J[1]

P01 <- P_0J[2]

P02 <- P_0J[3]

P03 <- P_0J[4]

P04 <- P_0J[5]

P05 <- P_0J[6]

# Divers parameters

comp_trans <- P_0J[2] + P_0J[5] + P_0J[6]

comp_dis_noTrans <- P_0J[3]

comp_death_noTrans <- P_0J[4]

AM_t <- (P05 / (P01 + P04 + P05)) - (P03 / (P00 + P02 + P03))

PAF_t <- (((P03 + P05) - (P03 / (P00 + P02 + P03))) / (P03 + P05) )

dat_c <- c("time_t" = t,

"P00" = P00,

"P01" = P01,

"P02" = P02,

"P03" = P03,

"P04" = P04,

"P05" = P05,

"comp_trans" = comp_trans,

"comp_dis_noTrans" = comp_dis_noTrans,

"comp_death_noTrans" = comp_death_noTrans,

"AM_t" = AM_t,

"PAF_t" = PAF_t)

return(dat_c)

}

#---------------

# Function 2.

#---------------

transition_probs <- function(s = 0,

t_max = 100,

Lambda_01,

Lambda_02,

Lambda_03,

Lambda_14,

Lambda_15) {

#create an artificial data.frame to save the computed rows into it:

dat_df <- data.frame("time_t" = s:t_max,

"P00" = s:t_max,

"P01" = s:t_max,

"P02" = s:t_max,

"P03" = s:t_max,

"P04" = s:t_max,

"P05" = s:t_max,

"comp_trans" = s:t_max,

"comp_dis_noTrans" = s:t_max,

"comp_death_noTrans" = s:t_max,

"AM_t" = s:t_max,

"PAF_t" = s:t_max)

# calculation of transition probabilities:

for (i in seq_along(s:t_max)) {

dat_df[i, ] <- transition_prob(t = (s:t_max)[i],

Lambda_01 = Lambda_01,

Lambda_02 = Lambda_02,

Lambda_03 = Lambda_03,

Lambda_14 = Lambda_14,

Lambda_15 = Lambda_15)

}

# the results:

return(dat_df)

}

#-----------------------------------------------------------------------------

# stacked plot function for eidm

#-----------------------------------------------------------------------------

stacked_plot_eidm <- function(s = 0,

t_max = 100,

x_lim = 100,

plot_title = "Stacked Probability Plot",

legend_position = c(0.8, 0.85),

order = c(2, 3, 0, 5, 4, 1), # oder of stacked plot from top to bottom

Lambda_01 , # initial - intermediate

Lambda_02 , # initial - discharge

Lambda_03 , # initial - death

Lambda_14 , # intermediate - discharge

Lambda_15 , # intermediate - death

area_col = c("khaki1", # inpatient w/o intermediate

"indianred1", # intermediate inpatient

"cornflowerblue", # discharge w/o intermediate

"darkblue", # death w/o intermediate

"chocolate", # discharge after intermediate

"chocolate4"), # death after intermediate

legend_labels = c("inpatient w/o intermediate",

"intermediate",

"discharge w/o intermediate",

"death w/o intermediate",

"discharge after intermediate",

"death after intermediate")) {

# transition probabilities

trans_prob <- transition_probs(s = 0,

t_max = t_max,

Lambda_01 = Lambda_01,

Lambda_02 = Lambda_02,

Lambda_03 = Lambda_03,

Lambda_14 = Lambda_14,

Lambda_15 = Lambda_15)

# stacket probabilities

line1 <- trans_prob[, paste0("P0", order[6])]

line2 <- line1 + trans_prob[, paste0("P0", order[5])]

line3 <- line2 + trans_prob[, paste0("P0", order[4])]

line4 <- line3 + trans_prob[, paste0("P0", order[3])]

line5 <- line4 + trans_prob[, paste0("P0", order[2])]

# plot those stacked probabilities

hui <- trans_prob[trans_prob$time_t >= 0, ] %>%

ggplot(aes()) +

ggtitle(plot_title) +

geom_ribbon(fill = area_col[order[6] + 1],

linetype = 0,

mapping = aes(x = time_t,

ymin = 0,

ymax = line1,

colour = "1")) +

geom_ribbon(fill = area_col[order[5] + 1],

linetype = 0,

mapping = aes(x = time_t,

ymin = line1,

ymax = line2,

colour = "2")) +

geom_ribbon(fill = area_col[order[4] + 1],

linetype = 0,

mapping = aes(x = time_t,

ymin = line2,

ymax = line3,

colour = "3")) +

geom_ribbon(fill = area_col[order[3] + 1],

linetype = 0,

mapping = aes(x = time_t,

ymin = line3,

ymax = line4,

colour = "4")) +

geom_ribbon(fill = area_col[order[2] + 1],

linetype = 0,

mapping = aes(x = time_t,

ymin = line4,

ymax = line5,

colour = "5")) +

geom_ribbon(fill = area_col[order[1] + 1],

linetype = 0,

mapping = aes(x = time_t,

ymin = line5,

ymax = 1,

colour= "6")) +

xlim(0, x_lim) +

theme(legend.position = legend_position,

legend.key.size = unit(3.2, "mm"),

legend.title = element_text(size = 16), #change legend title font size

legend.text = element_text(size = 14),

legend.background = element_rect(fill = "grey85",

size = 1,

linetype = "solid")) +

scale_color_manual(labels = legend_labels[order + 1],

values = area_col[order + 1]) +

ylab("Probability") +

xlab("follow-up time") +

guides(colour = guide_legend(override.aes = list(fill = area_col[order + 1]))) +

labs(col="Events")

return_list <- list()

return_list[[1]] <- trans_prob

return_list[[2]] <- hui

return(return_list)

}

#-----------------------------------------------------------------------------

# Combine stacked plots, PAF and AM

#-----------------------------------------------------------------------------

eidm_aggregated_hazards <- function(s = 0,

t_max = 100,

x_lim = 100,

legend_position_eidm = c(0.8, 0.85),

plot_title_eidm = "Stacked Probability Plot",

order_eidm = c(2, 3, 0, 5, 4, 1),

Lambda_01,

Lambda_02,

Lambda_03,

Lambda_14,

Lambda_15,

area_col_eidm = c("cornflowerblue", # discharge w/o intermediate

"darkblue", # death w/o intermediate

"khaki1", # inpatient w/o intermediate

"chocolate4", # death after intermediate

"chocolate", # discharge after intermediate

"indianred1"), # intermediate inpatient

legend_labels_eidm = c("discharge w/o intermediate",

"death w/o intermediate",

"inpatient w/o intermediate",

"death after intermediate",

"discharge after intermediate",

"intermediate"),

paf_title = "PAF(t)",

am_title = "AM(t)") {

hui_eidm <- stacked_plot_eidm(s = 0,

t_max = t_max,

x_lim = x_lim,

Lambda_01 , # initial - intermediate

Lambda_02 , # initial - discharge

Lambda_03 , # initial - death

Lambda_14 , # intermediate - discharge

Lambda_15 , # intermediate - death

order = order_eidm,

area_col = area_col_eidm,

plot_title = plot_title_eidm,

legend_position = legend_position_eidm,

legend_labels = legend_labels_eidm)

#----------

# PAF_t

#----------

p1 <- ggplot(hui_eidm[[1]][hui_eidm[[1]]$time_t > 0,], # at t = 0 PAF is NA as each Pij is zero

aes(time_t,

PAF_t)) +

geom_area(aes(time_t,

PAF_t)) +

# geom_hline(yintercept = 0) +

ggtitle(paf_title) +

xlim(ifelse(s == 0, 0, s), x_lim)

#----------

# AM_t

#----------

p2 <- ggplot(hui_eidm[[1]][hui_eidm[[1]]$time_t>0,], # at t = 0 AM is NA as each Pij is zero

aes(time_t,

AM_t)) +

geom_area(aes(time_t,

AM_t)) +

# geom_hline(yintercept = 0) +

xlim(ifelse(s == 0, 0, s), x_lim) +

ggtitle(am_title)

results_list <- list()

results_list[[1]] <- hui_eidm[[1]]

results_list[[2]] <- hui_eidm[[2]]

results_list[[3]] <- p1

results_list[[4]] <- p2

names(results_list) <- c("transition Probabilities",

"stacked plot - eidm",

"PAF(t)",

"AM(t)")

return(results_list)

}

# extract legend in order to obtain one legend.

g_legend <- function(a.gplot){

tmp <- ggplot_gtable(ggplot_build(a.gplot))

leg <- which(sapply(tmp$grobs, function(x) x$name) == "guide-box")

legend <- tmp$grobs[[leg]]

return(legend)

}

## interface.R

#-------------------------------------------------------------------------------

#

# Script for generating the Interface and overall appearance

#

# Copyright (C) 2023 Marlon Grodd, Susanne Weber, Martin Wolkewitz

#

# This program is free software: you can redistribute it and/or modify

# it under the terms of the GNU General Public License as published by

# the Free Software Foundation, either version 3 of the License, or

# (at your option) any later version.

#

# This program is distributed in the hope that it will be useful,

# but WITHOUT ANY WARRANTY; without even the implied warranty of

# MERCHANTABILITY or FITNESS FOR A PARTICULAR PURPOSE. See the

# GNU General Public License for more details.

#

# You should have received a copy of the GNU General Public License

# along with this program. If not, see <http://www.gnu.org/licenses/>.

#

#-------------------------------------------------------------------------------

# Define UI for application

ui <- fluidPage(

div(HTML('<img src="combacte_logo.png" width = 240 alt="COMBACTE logo" style="margin-top: 25px">

<img src="ukl_logo.png" width = 240 alt="ukl logo" style="float:right;margin-top: 35px">

<img src="fdm_logo.png" width = 240 alt="fdm logo" style="float:right">')),

# div(img(src = "combacte_logo.jpg",

# width = 240,

# alt = "COMBACTE Logo")),

# fluidRow(

# column(1,

# tags$img(src = "combacte_logo.jpg",

# width = 240,

# alt = "COMBACTE Logo"),

# tags$img(src = "fdm_logo.png",

# width = 240,

# alt = "COMBACTE Logo"),

# tags$img(src = "ukl_logo.png",

# width = 240,

# alt = "COMBACTE Logo"))

# ),

# Banner

# tags$figure(

# align = "left",

# tags$img(src = "combacte_logo.jpg",

# width = 240,

# alt = "COMBACTE Logo"),

#

# align = "right",

# tags$img(src = "fdm_logo.png",

# width = 240,

# alt = "COMBACTE Logo")),

#

# tags$figure(

# align = "right",

# tags$img(src = "ukl_logo.png",

# width = 240,

# alt = "COMBACTE Logo")),

# Application title

titlePanel("Extended illness-death model with constant transition hazards"),

div(HTML('This is a tool to visualize time-dependend effects in a multi-state-setting considering an extended illness-death model with constant hazards.<br>

<br>

<ul>

<li>Two groups can be compared (Group A and Group B)</li>

<li>Hazards can be provided in two ways:<br>

<ul style="list-style-type:square;">

<li>Explicit for each group (Group A and B)</li>

<li>Baseline Hazards (Group A) and hazard ratios</li>

</ul>

</li>

<li>Description of items:<br>

<ul style="list-style-type:square;">

<li>"limit of x-axis": set the time horizon</li>

<li>"order for stacked plot": order of the ploted areas in the stacked probability plots</li>

<li>"Show PAF plot": Render the plot the population attributable fraction</li>

<li>"Show PAF plot": Render the plot the attributable mortality</li>

</ul>

</li>

<li>Formula for Hazards:</li>')),

tags$figure(

align = "center",

tags$img(src = "hazard.png",

width = 300,

alt = "Formula for Hazards")),

br(),

# create one row with input and output:

fluidRow(

#allow for two input options

navbarPage("Group Hazards or Hazard ratio?",

#, create two separate tabPanels for each input option

tabPanel("Group Hazards",

fluidRow(column(width=3,

fluidRow(

column(width = 6,

"Group A",

numericInput("A01_a", "\u03BB\u2080\u2081", 0.05),

numericInput("A02_a", "\u03BB\u2080\u2082", 0.1),

numericInput("A03_a", "\u03BB\u2080\u2083", 0.05),

numericInput("A14_a", "\u03BB\u2081\u2084", 0.1),

numericInput("A15_a", "\u03BB\u2081\u2085", 0.05)

),

column(width = 6,

"Group B",

numericInput("A01_b", "\u03BB\u2080\u2081", 0.05),

numericInput("A02_b", "\u03BB\u2080\u2082", 0.1),

numericInput("A03_b", "\u03BB\u2080\u2083", 0.05),

numericInput("A14_b", "\u03BB\u2081\u2084", 0.1),

numericInput("A15_b", "\u03BB\u2081\u2085", 0.05)

)),

fluidRow(column(width = 12,

numericInput("x_lim", "limit of x-axis", 30),

textInput("order",

"order for stacked plot from top to bottom according to number of state in eidm

(enter a vector (comma delimited))", "2,3,0,5,4,1"),

checkboxInput("PAF", "Show PAF plot?", FALSE),

checkboxInput("AM", "Show AM plot?", FALSE),

))),

column(9,

tags$figure(

align = "center",

tags$figcaption("The extended illness death model with constant transition hazards"),

tags$img(

src = "eidm.png",

width = 600,

alt = "The extended illness death model with constant transition hazards")),

br(),

br(),

plotOutput("stackedPlot2"),

plotOutput("stackedPlot2a", height = "100px"),

plotOutput("stackedPlot3"),

plotOutput("stackedPlot4"))))

,

tabPanel("Hazard ratio",

fluidRow(column(width = 3,

fluidRow(

column(width = 6,

"Baseline",

numericInput("A01_c", "\u03BB\u2080\u2081", 0.05),

numericInput("A02_c", "\u03BB\u2080\u2082", 0.1),

numericInput("A03_c", "\u03BB\u2080\u2083", 0.05),

numericInput("A14_c", "\u03BB\u2081\u2084", 0.1),

numericInput("A15_c", "\u03BB\u2081\u2085", 0.05)

),

column(width = 6,

"Hazard ratio",

numericInput("A01_hr", "HR\u2080\u2081", 2),

numericInput("A02_hr", "HR\u2080\u2082", 1),

numericInput("A03_hr", "HR\u2080\u2083", 1),

numericInput("A14_hr", "HR\u2081\u2084", 1),

numericInput("A15_hr", "HR\u2081\u2085", 0.5)

)),

fluidRow(column(width = 12,

numericInput("x_lim_hr", "limit of x-axis", 30),

textInput("order_hr", "order for stacked plot from top to bottom according to number of state in eidm

(enter a vector (comma delimited))", "2,3,0,5,4,1"),

checkboxInput("PAF_hr", "Show PAF plot?", FALSE),

checkboxInput("AM_hr", "Show AM plot?", FALSE),

))),

column(9,

tags$figure(

align = "center",

tags$figcaption("The extended illness death model with constant transition hazards"),

tags$img(

src = "eidm.png",

width = 600,

alt = "The extended illness death model with constant transition hazards")),

br(),

br(),

plotOutput("stackedPlot2_hr"),

plotOutput("stackedPlot2a_hr", height = "100px"),

plotOutput("stackedPlot3_hr"),

plotOutput("stackedPlot4_hr")))))))

# Define server in order to use input to create output

server <- function(input, output) {

# for easy use a short version

eidm_aggregated_hazards_short <- function(s = 0,

t_max = input$x_lim,

x_lim = input$x_lim,

legend_position_eidm,

plot_title_eidm = "Stacked Probability Plot",

order_eidm,

Lambda_01,

Lambda_02,

Lambda_03,

Lambda_14,

Lambda_15,

area_col_eidm = c("khaki1", # inpatient w/o intermediate

"indianred1", # intermediate inpatient

"cornflowerblue", # discharge w/o intermediate

"darkblue", # death w/o intermediate

"chocolate", #discharge after intermediate

"chocolate4"), # death after intermediate

legend_labels_eidm = c("inpatient w/o intermediate",

"intermediate",

"discharge w/o intermediate",

"death w/o intermediate",

"discharge after intermediate",

"death after intermediate"),

paf_title = "PAF(t)",

am_title = "AM(t)") {

eidm_aggregated_hazards(s = s,

t_max = t_max,

x_lim = x_lim,

legend_position_eidm = legend_position_eidm,

plot_title_eidm = plot_title_eidm,

order_eidm = order_eidm,

Lambda_01 = Lambda_01,

Lambda_02 = Lambda_02,

Lambda_03 = Lambda_03,

Lambda_14 = Lambda_14,

Lambda_15 = Lambda_15,

area_col_eidm = area_col_eidm,

legend_labels_eidm = legend_labels_eidm,

paf_title = paf_title,

am_title = am_title)

}

# stacked plot group A and B

output$stackedPlot2 <- renderPlot({

order_vector <- as.numeric(unlist(strsplit(input$order, ",")))

hui_a <- eidm_aggregated_hazards_short(legend_position_eidm = "bottom",

plot_title_eidm = "Stacked Probability Plot Group A",

order_eidm = order_vector,

Lambda_01 = input$A01_a,

Lambda_02 = input$A02_a,

Lambda_03 = input$A03_a,

Lambda_14 = input$A14_a,

Lambda_15 = input$A15_a)

hui_b <- eidm_aggregated_hazards_short(legend_position_eidm = c(0.8, 0.85),

plot_title_eidm = "Stacked Probability Plot Group A",

order_eidm = order_vector,

Lambda_01 = input$A01_b,

Lambda_02 = input$A02_b,

Lambda_03 = input$A03_b,

Lambda_14 = input$A14_b,

Lambda_15 = input$A15_b)

# draw plots without legend:

grid.arrange(hui_a[[2]] + theme(legend.position = "none"),

hui_b[[2]] + theme(legend.position = "none"), nrow = 1)

})

# separate legend Plot for EIDM:

output$stackedPlot2a <- renderPlot({

order_vector <- as.numeric(unlist(strsplit(input$order, ",")))

hui_a <- eidm_aggregated_hazards_short(legend_position_eidm = "top",

order_eidm = order_vector,

Lambda_01 = input$A01_a,

Lambda_02 = input$A02_a,

Lambda_03 = input$A03_a,

Lambda_14 = input$A14_a,

Lambda_15 = input$A15_a)

#get legend from one plot:

mylegend <- g_legend(hui_a[[2]])

# draw plots with one combined legend:

grid.arrange(mylegend)

})

# PAF plot for group A and B

output$stackedPlot3 <- renderPlot({

order_vector <- as.numeric(unlist(strsplit(input$order, ",")))

hui_a <- eidm_aggregated_hazards_short(legend_position_eidm = c(0.8, 0.85),

order_eidm = order_vector,

Lambda_01 = input$A01_a,

Lambda_02 = input$A02_a,

Lambda_03 = input$A03_a,

Lambda_14 = input$A14_a,

Lambda_15 = input$A15_a)

hui_b <- eidm_aggregated_hazards_short(legend_position_eidm = c(0.8, 0.85),

order_eidm = order_vector,

Lambda_01 = input$A01_b,

Lambda_02 = input$A02_b,

Lambda_03 = input$A03_b,

Lambda_14 = input$A14_b,

Lambda_15 = input$A15_b)

# plot only if input box is TRUE

if(input$PAF == TRUE){

grid.arrange(hui_a[[3]],

hui_b[[3]],

nrow = 1) }

})

# AM plot for group A and B

output$stackedPlot4 <- renderPlot({

order_vector <- as.numeric(unlist(strsplit(input$order, ",")))

hui_a <- eidm_aggregated_hazards_short(legend_position_eidm = c(0.8, 0.85),

order_eidm = order_vector,

Lambda_01 = input$A01_a,

Lambda_02 = input$A02_a,

Lambda_03 = input$A03_a,

Lambda_14 = input$A14_a,

Lambda_15 = input$A15_a)

hui_b <- eidm_aggregated_hazards_short(legend_position_eidm = c(0.8, 0.85),

order_eidm = order_vector,

Lambda_01 = input$A01_b,

Lambda_02 = input$A02_b,

Lambda_03 = input$A03_b,

Lambda_14 = input$A14_b,

Lambda_15 = input$A15_b)

# plot only if input box is TRUE

if(input$AM == TRUE){

grid.arrange(hui_a[[4]],

hui_b[[4]],

nrow = 1)}

})

#

# Similar for HR input:

#

output$stackedPlot2_hr <- renderPlot({

order_vector <- as.numeric(unlist(strsplit(input$order_hr, ",")))

a01_b <- input$A01_c * input$A01_hr

a02_b <- input$A02_c * input$A02_hr

a03_b <- input$A03_c * input$A03_hr

a14_b <- input$A14_c * input$A14_hr

a15_b <- input$A15_c * input$A15_hr

hui_a <- eidm_aggregated_hazards_short(t_max = input$x_lim_hr,

x_lim = input$x_lim_hr,

legend_position_eidm = "bottom",

order_eidm = order_vector,

Lambda_01 = input$A01_c,

Lambda_02 = input$A02_c,

Lambda_03 = input$A03_c,

Lambda_14 = input$A14_c,

Lambda_15 = input$A15_c)

hui_b <- eidm_aggregated_hazards_short(t_max = input$x_lim_hr,

x_lim = input$x_lim_hr,

legend_position_eidm = c(0.8, 0.85),

order_eidm = order_vector,

Lambda_01 = a01_b,

Lambda_02 = a02_b,

Lambda_03 = a03_b,

Lambda_14 = a14_b,

Lambda_15 = a15_b)

# draw plots without legend:

grid.arrange(hui_a[[2]] + theme(legend.position = "none"),

hui_b[[2]] + theme(legend.position = "none"), nrow = 1)

})

output$stackedPlot2a_hr <- renderPlot({

order_vector <- as.numeric(unlist(strsplit(input$order_hr, ",")))

a01_b <- input$A01_c * input$A01_hr

a02_b <- input$A02_c * input$A02_hr

a03_b <- input$A03_c * input$A03_hr

a14_b <- input$A14_c * input$A14_hr

a15_b <- input$A15_c * input$A15_hr

hui_a <- eidm_aggregated_hazards_short(t_max = input$x_lim_hr,

x_lim = input$x_lim_hr,

legend_position_eidm = "top",

order_eidm = order_vector,

Lambda_01 = input$A01_c,

Lambda_02 = input$A02_c,

Lambda_03 = input$A03_c,

Lambda_14 = input$A14_c,

Lambda_15 = input$A15_c)

#get legend from one plot:

mylegend <- g_legend(hui_a[[2]])

# draw plots with one combined legend:

grid.arrange(mylegend)

})

output$stackedPlot3_hr <- renderPlot({

order_vector <- as.numeric(unlist(strsplit(input$order_hr, ",")))

a01_b <- input$A01_c * input$A01_hr

a02_b <- input$A02_c * input$A02_hr

a03_b <- input$A03_c * input$A03_hr

a14_b <- input$A14_c * input$A14_hr

a15_b <- input$A15_c * input$A15_hr

hui_a <- eidm_aggregated_hazards_short(t_max = input$x_lim_hr,

x_lim = input$x_lim_hr,

legend_position_eidm = c(0.8, 0.85),

order_eidm = order_vector,

Lambda_01 = input$A01_c,

Lambda_02 = input$A02_c,

Lambda_03 = input$A03_c,

Lambda_14 = input$A14_c,

Lambda_15 = input$A15_c)

hui_b <- eidm_aggregated_hazards_short(t_max = input$x_lim_hr,

x_lim = input$x_lim_hr,

legend_position_eidm = c(0.8, 0.85),

order_eidm = order_vector,

Lambda_01 = a01_b,

Lambda_02 = a02_b,

Lambda_03 = a03_b,

Lambda_14 = a14_b,

Lambda_15 = a15_b)

# plot only if input box is TRUE

if(input$PAF_hr == TRUE){

grid.arrange(hui_a[[3]],

hui_b[[3]],

nrow = 1) }

})

output$stackedPlot4_hr <- renderPlot({

order_vector <- as.numeric(unlist(strsplit(input$order_hr, ",")))

a01_b <- input$A01_c * input$A01_hr

a02_b <- input$A02_c * input$A02_hr

a03_b <- input$A03_c * input$A03_hr

a14_b <- input$A14_c * input$A14_hr

a15_b <- input$A15_c * input$A15_hr

hui_a <- eidm_aggregated_hazards_short(t_max = input$x_lim_hr,

x_lim = input$x_lim_hr,

legend_position_eidm = c(0.8, 0.85),

order_eidm = order_vector,

Lambda_01 = input$A01_c,

Lambda_02 = input$A02_c,

Lambda_03 = input$A03_c,

Lambda_14 = input$A14_c,

Lambda_15 = input$A15_c)

hui_b <- eidm_aggregated_hazards_short(t_max = input$x_lim_hr,

x_lim = input$x_lim_hr,

legend_position_eidm = c(0.8, 0.85),

order_eidm = order_vector,

Lambda_01 = a01_b,

Lambda_02 = a02_b,

Lambda_03 = a03_b,

Lambda_14 = a14_b,

Lambda_15 = a15_b)

# plot only if input box is TRUE

if(input$AM_hr == TRUE){

grid.arrange(hui_a[[4]],

hui_b[[4]],

nrow = 1)}

})

}
